# Supplementary material for: cfr and fexA genes in methicillin-resistant Staphylococcus aureus from humans and livestock in the Netherlands
Source: Commun Med (Lond). 2022 Oct 28;2:135. doi: 10.1038/s43856-022-00200-w (PMC9616846; doi:10.1038/s43856-022-00200-w)
Supplement: Supplementary file 1 — Supplementary information [file 43856_2022_200_MOESM1_ESM.pdf]

Supplementary information

***cfr* and *fexA* genes in methicillin-resistant *Staphylococcus aureus* from humans and livestock in the Netherlands**

Leo M. Schouls, Kees Veldman, Michael S.M. Brouwer, Cindy Dierikx, Sandra Witteveen, Marga van Santen-Verheuvel, Antoni P.A. Hendrickx, Fabian Landman, Paul Hengeveld, Bart Wullings, Michel Rapallini, Ben Wit, Engeline van Duijkeren and the Dutch MRSA surveillance study group.

Supplementary Table 1.

Distribution of clonal complexes (CCs) of isolates obtained from humans in the national MRSA surveillance for which next-generation sequencing data was available.

CC or ST	2003	2004	2005	2006	2007	2008	2009	2010	2011	2012	2013	2014	2015	2016	2017	2018	2019	2020	2021	All years	%
CC1														9	69	59	118	97	26	378	6
CC5						3		5	7	6	5	6	18	78	158	144	231	205	38	904	14
CC6														2	23	35	71	75	24	230	4
CC7															3	3	11	2		19	0.3
CC8							3	4	4	8	6	2	8	57	154	155	189	188	26	804	13
CC9														1	2	1	6	5		15	0.2
CC12															1					1	0.02
CC15															4	5	18	27	8	62	1
CC22						1					4	12	19	43	131	94	225	117	16	662	10
CC30												1		14	95	83	98	74	4	369	6
CC59														9	37	30	31	26	5	138	2
CC72														1	19	19	38	39	2	118	2
CC80														3	27	11	29	18	3	91	1
CC88														4	32	21	34	42	6	139	2
CC93															1	5	2			8	0.1
CC96																2		2	1	5	0.1
CC97														4	22	11	33	29	5	104	2
CC121												1		1	2	7	30	9		50	1
CC130						2	2	1	5	3	4	3	5	6	6	6	7	5	1	56	1
CC140																			1	1	0.02
CC152														1	13	14	16	16	1	61	1
CC188														2	2	8	14	12		38	1
ST207																1				1	0.02
CC361															1	10	12	11	3	37	1
CC398	14	14	10	17	29	40	80	82	65	65	17	36	63	97	162	158	321	318	53	1,641	26
ST718															3			1		4	0.1
CC772														2	13	12	19	17	2	65	1
CC779																1	3	2		6	0.1
ST834															1	2		2		5	0.1
CC913															1	1		3	2	7	0.1
CC45									18	8				14	36	26	11	6	1	120	2
CC1153																2		7		9	0.1
ST1162														1						1	0.02
CC1420																		1		1	0.02
CC1943										1	1				4		1	1		8	0.1
CC45								2	2					19	28	24	38	50	5	168	3
ST5116																1				1	0.02
Total	14	14	10	17	29	46	85	94	101	91	37	61	113	368	1050	951	1606	1407	233	6,327	

CCs are based on the ST and its CC classification as reported in literature. If no CC was available, the sequence type (ST) is provided. %, percentage of all isolates.

Supplementary Table 2.

CC398 isolates from animals, animal farms or slaughterhouses and from persons working in these facilities.

Animal species	Num. of isolates per species	Specimen type	Sampling year 2001	2004	2005	2007	2008	2010	2011	2013	2014	2015	2018	2019	unknown	All years
Cattle	22	Swab							21							21
		Dust slaughterhouse					1									1
Duck	2	Throat								2						2
Human	14	Nose						9	1				1		3	14
Pig	218	Nose				23						112				135
		Dust farm				1								57		58
		Meat											2	12		14
		Feces	1	5	1		4									11
Broiler	54	Meat broiler											23			23
		Throat at slaughterhouse					12									12
		Dust slaughterhouse					10									10
		Dust farm						4								4
		Throat						4								4
		Feces						1								1
Turkey	22	Dust farm									9					9
		Nose									7					7
		Meat											3			3
		Throat									3					3
All species	332		1	5	1	24	27	18	22	2	19	112	29	69	3	332

Supplementary Table 3.

Minimum inhibitory concentrations (MICs, in mg/L) as assessed by microbroth dilution assays using two panels: European panel (EUST) and Custom-made panel (NLD1GNS).

	European Panel (EUST) CHL	CIP	CLI	ERY	FOX	FUS	GEN	KAN	LZD	MUP	PEN	RIF	SMX	STR	SYN	TET	TIA	TMP	VAN	Custom made panel (NLD1GNS) CEQ*	DOX	ENRO**	FFN	GEN	KAN	NEO	SXT	TET	XNL*
H1	**64**	**8**	**>4**	**>8**	**16**	≤0.5	≤1	≤4	4	≤0.5	**>2**	≤0.016	≤64	**>32**	**>4**	**>16**	**>4**	**>32**	≤1	**>0.5**	**16**	**>2**	**>32**	0.25	4	**8**	**0.5/9.5**	**>32**	**>0.5**
H2	**>64**	≤0.25	**>4**	≤0.25	**8**	≤0.5	≤1	≤4	**8**	≤0.5	**>2**	≤0.016	≤64	8	**4**	**>16**	**>4**	≤2	≤1	**>0.5**	**16**	0.12	**>32**	0.25	1	0.25	0.06/1.19	**>32**	**>0.5**
H3	**>64**	≤0.25	**>4**	>8	**16**	≤0.5	≤1	≤4	**8**	≤0.5	**>2**	≤0.016	≤64	16	**>4**	**>16**	**>4**	≤2	≤1	**>0.5**	**16**	0.12	**>32**	0.25	2	1	0.12/2.38	**>32**	**>0.5**
H4	**>64**	**>8**	**>4**	≤0.25	**8**	≤0.5	≤1	8	**8**	≤0.5	**2**	≤0.016	≤64	≤4	**2**	**>16**	**>4**	**>32**	≤1	**>0.5**	**16**	**>2**	**>32**	0.25	8	**8**	**2/38**	**>32**	**>0.5**
H5	**64**	≤0.25	≤0.12	0.5	**16**	≤0.5	2	**16**	≤1	≤0.5	**>2**	≤0.016	≤64	16	≤0.5	**>16**	1	**>32**	≤1	**>0.5**	**8**	0.12	**>32**	1	**32**	1	**0.5/9.5**	**>32**	**>0.5**
H6	**>64**	**2**	**>4**	≤0.25	**8**	≤0.5	≤1	≤4	**8**	≤0.5	**>2**	≤0.016	≤64	8	**2**	**>16**	**>4**	**>32**	≤1	**>0.5**	**16**	**1**	**>32**	0.25	1	0.25	0.25/4.75	**>32**	**>0.5**
H7	**>64**	≤0.25	≤0.12	0.5	**>16**	≤0.5	≤1	≤4	2	≤0.5	**2**	≤0.016	≤64	8	≤0.5	**>16**	≤0.5	**>32**	≤1	**>0.5**	**8**	0.12	**>32**	0.5	2	0.5	**0.5/9.5**	**>32**	**>0.5**
P1	**>64**	≤0.25	**>4**	≤0.25	**16**	≤0.5	≤1	≤4	**8**	≤0.5	**>2**	≤0.016	≤64	**>32**	**2**	**>16**	**>4**	**>32**	≤1	**>0.5**	**16**	0.12	**>32**	0.12	1	0.25	**0.5/9.5**	**>32**	**>0.5**

*MICs of resistant isolates are in bold. *No EUCAST ECOFF, **CSLI breakpoints used.*

List of abbreviations of the antibiotics used in the MIC analyses

Abbreviation	Antibiotic
CEQ	cefquinome*
CHL	chlooramfenicol
CIP	ciprofloxacin
CLI	clindamycin
DOX	doxycycline
ENRO	enrofloxacin**
ERY	erythromycin
FFN	florfenicol
FOX	cefoxitin
FUS	fusidinic acid
GEN	gentamicin
KAN	kanamycin
LZD	linezolid

Abbreviation	Antibiotic
MUP	mupirocin
NEO	neomycin
PEN	(benzyl)penicillin
RIF	rifampicin
SMX	sulfamethoxazole
STR	streptomycine
SXT	trimethoprim/sulfamethoxazole
SYN	quinupristin/dalfopristin (synercid)
TET	tetracycline
TIA	tiamulin
TMP	trimethoprim
VAN	vancomycin
XNL	ceftiofur*

Supplementary Table 4.

Resistance genes in the genomes of eight *cfr*-positive isolates and their similarity with entries in the NCBI database (nt/nr).

		Resistance genes																				Closest matching plasmid in NCBI database (BLAST)				
Isolate	Size (bp)	*cfr*	*fexA*	*aac(6')-aph(2'')*	*aadD*	*ant(6)-Ia*	*ant(9)-Ia*	*blaZ*	*mecA*	*dfrG*	*dfrK*	*erm(B)*	*erm(C)*	*lnu(A)*	*lnu(B)*	*lsa(B)*	*lsa(E)*	*str*	*tet(K)*	*tet(L)*	*tet(M)*	Acc.num.	Plasmid	% Identity/Coverage	Species	Size (bp)
H1 chromosome	2,933,086		19		■	■		■	■	■					■		■		■		■					
H1 plasmid 1	13,757	■										■								■		MH431899	Sch-734Lar	100/80	*S. chromogenes*	10,252
H2 chromosome	2,806,671	■	20					■	■										■		■					
H3 chromosome	2,844,988		14					■	■										■		■					
H3 plasmid 1	38,430	■	14																			CP065195	Unnamed	98/95	LA-MRSA	37,510
H3 plasmid 2	2,997																					CP013626	pRIVM4296	>99/100	LA-MRSA	2,992
H3 plasmid 3	2,407												■									MN251860	pSR383	>99/100	*S. aureus*	2,413
H4 chromosome	2,893,482							■	■		■								■		■					
H4 plasmid 1	39,628	■	21		■						■					■				■		JN970906*	pERGB	>99/38	*S. aureus*	15,259
H4 plasmid 2	2,361													■								CP014694	plinE154	>99/100	LA-MRSA	2,361
H5 chromosome	2,890,216			▒				■	■		■										■					
H5 plasmid 1	37,004	▒	23																			CP065195	Unnamed	97/93	LA-MRSA	37,510
H6 chromosome	2,795,636							■	■		■								■		■					
H6 plasmid 1	35,612	■	20																			MH660279	p0915	92/65	*S. aureus*	35,004
H7 chromosome	2,817,149							■	■		■										■					
H7 plasmid 1	34,523	▒	13																			KM521837	p12-02300	>99/26	*S. epidermidis*	38,864
P1 chromosome	2,796,375						■	■	■	■					■		■		■		■					
P1 plasmid 1	37,279	■	11																			CP065195	Unnamed	98/88	LA-MRSA	37,510
P1 plasmid 2	4,397																	■				CP076841	Unnamed	>99/100	LA-MRSA	4,397
P1 plasmid 3	3,971																					CP049804	Unnamed	91/89	*S. warneri*	3,919

The black boxes indicate the presence of the resistance genes. The gray boxes indicate the gene is inactive due to mutation or deletion of a single nucleotide. The numbers in the black boxes denote the *fexA* allele. *This plasmid also has high degree of similarity with KX520649, the p14-01514 plasmid from *S. epidermidis*.

Supplementary Table 5.

Distribution of *fexA* sequence variants among clonal complexes of the study set.

	National MRSA surveillance												CC398 isolates from livestock sampling				
CCs	CC5				CC6	CC8	CC9	CC59	CC398				Human nose	Pig farm dust	Pig meat		Pig nose
N	904				230	804	15	138	1,641				14	58	14		14
fexA variant	ST4593	ST5	ST5258	ST5599	ST6	ST8	ST9	ST59	ST398	ST4159	ST5857	ST7191	ST398	ST398	ST398	ST5972	ST398
fexA01									10					1			3
fexA03									70					8	1	1	3
fexA04									1								
fexA05									18		2						
fexA06									3								
fexA07									2				1				
fexA08									2								
fexA09									1								
fexA10									1								
fexA11									4	1				1			
fexA12					1												
fexA13									8					1			
fexA14									1								
fexA15							1										
fexA16									2			1					3
fexA17	1	76	1	1													
fexA19									10					2			
fexA20									2								
fexA21									1								
fexA22									1								
fexA23									8								
fexA24		1															
fexA25		2															
fexA27		1															
fexA28		3															
fexA29		1															
fexA30								1									
fexA31		1															
fexA32		1															
fexA33		2															
fexA34						3											
All *fexA* variants	1	88	1	1	1	3	1	1	145	1	2	1	1	13	2		9

The isolates from the pig fecal samples (*n*=11), cattle samples (*n*=22) and poultry samples (*n*=78) did not carry a *fexA* gene. *N*, number of isolates per sample category.

Supplementary Table 6.

Distribution of *fexA* variants found among the NCBI database entries (nr/nt).

	Staphylococcus(-like)			Other genera															
fexA variant	*Staphylococcus aureus*	Other *Staphylococcus spp.*	*Mammalicoccus*	*Enterococcus*	*Campylobacter*	*Lactococcus*	*Bacillus*	*Streptococcus*	*Listeria*	*Vagococcus*	*Fusobacterium*	*Klebsiella*	*Limosilactobacillus*	*Lactobacillus*	*Hafnia*	*Clostridium*	*Exiguobacterium*	*Pseudomonas*	Total
fexA01	5	3	6	1															15
fexA02		2																	2
fexA03		3													1				4
fexA06				1															1
fexA07	1																		1
fexA08	1						1												2
fexA11		1		1															2
fexA12							3												3
fexA13				1															1
fexA17	2																		2
fexA19	2																		2
fexA22	1	1	5	11				1	2										21
fexA34	1	1																	2
fexA35		5		75	5	3		3		1	1					1			94
fexA36				2															2
fexA37		2	1																3
fexA38			4																4
fexA39						4													4
fexA40						4													4
fexA42																	1		1
fexA43												1							1
fexA44					10														10
fexA45													1						1
fexA47	1																		1
fexA48		3	2																5
fexA51				13															13
fexA52				1															1
fexA53		1																	1
fexA54	1																		1
fexA55	1																		1
fexA56	2																		2
fexA57				1															1
fexA58														1					1
fexA59	1																		1
fexA60		1																	1
fexA63					1														1
fexA64			1																1
fexA65									1										1
fexA66				1															1
fexA67	1																		1
fexA68	1																		1
fexA69		1																	1
fexA70		1																	1
fexA72				1															1
fexA73				1															1
fexA74			8																8
fexA75				1															1
fexA76				1															1
fexA77																		1	1
fexA78			3	15															18
fexA79				1															1
fexA80	1			1															2
fexA81				1															1
fexA82	1																		1
fexA83		2																	2
All	23	27	30	130	16	11	4	4	3	1	1	1	1	1	1	1	1	1	257

The *fexA* sequence variants found in the study-isolates are displayed in bold.

Supplementary Table 7.

NCBI database entries (nr/nt) with a *fexA* gene with ≥98.7% nucleotide sequence identity with *fexA01*.

	Location			
Genus	Chromosome	Plasmid	Unknown	Total
Staphylococcus aureus	13	6	4	23
Other *Staphylococcus* spp.	9	12	6	27
Mammaliicoccus	6	7	17	30
Enterococcus	18	83	29	130
Campylobacter	14	1	1	16
Lactococcus	1	10		11
Bacillus	3		1	4
Streptococcus		1	3	4
Listeria	3			3
Vagococcus	1			1
Fusobacterium	1			1
Klebsiella		1		1
Limosilactobacillus		1		1
Lactobacillus	1			1
Hafnia			1	1
Clostridium		1		1
Exiguobacterium		1		1
Pseudomonas			1	1
All	70	124	63	257

Supplementary Table 8.

Minimum inhibitory concentrations for chloramphenicol (R>16 mg/L) and florfenicol (R>8 mg/L).

A.

				MIC	
Isolate	*fexA* variant	Source	CC (ST)	Chloramphenicol	Florfenicol
RIVM_M038386	*fexA01*	Human - Surveillance	CC398 (ST398)	64	32
RIVM_M047084	*fexA01*	Human - Surveillance	CC398 (ST398)	64	32
RIVM_M086974	*fexA01*	Human - Surveillance	CC398 (ST398)	64	32
RIVM_M085084	*fexA01*	Pig farm dust	CC398 (ST398)	64	>32
RIVM_M081140	*fexA01*	Pig nose	CC398 (ST398)	64	32
RIVM_M081142	*fexA01*	Pig nose	CC398 (ST398)	64	32
RIVM_M033294	*fexA03*	Human - Surveillance	CC398 (ST398)	>64	32
RIVM_M082615	*fexA03*	Human - Surveillance	CC398 (ST398)	64	32
RIVM_M087624	*fexA03*	Human - Surveillance	CC398 (ST398)	64	32
RIVM_M085078	*fexA03*	Pig farm dust	CC398 (ST398)	64	32
RIVM_M085099	*fexA03*	Pig farm dust	CC398 (ST398)	64	32
RIVM_M085127	*fexA03*	Pig meat	CC398 (ST398)	64	32
RIVM_M035021	*fexA05*	Human - Surveillance	CC398 (ST398)	64	>32
RIVM_M048322	*fexA05*	Human - Surveillance	CC398 (ST398)	32	32
RIVM_M085316	*fexA05*	Human - Surveillance	CC398 (ST398)	64	>32
RIVM_M043030	*fexA17*	Human - Surveillance	CC5 (ST5)	64	8
RIVM_M047957	*fexA17*	Human - Surveillance	CC5 (ST5)	64	4
RIVM_M084722	*fexA17*	Human - Surveillance	CC5 (ST5)	64	8
RIVM_M047665	*fexA19*	Human - Surveillance	CC398 (ST398)	64	>32
RIVM_M084839	*fexA19*	Human - Surveillance	CC398 (ST398)	32	>32
RIVM_M086341	*fexA19*	Human - Surveillance	CC398 (ST398)	32	32
RIVM_M041941	*fexA28*	Human - Surveillance	CC5 (ST5)	32	8
RIVM_M046661	*fexA28*	Human - Surveillance	CC5 (ST5)	32	8
RIVM_M047371	*fexA28*	Human - Surveillance	CC5 (ST5)	32	4

B.

	Amino acid position						
fexA allele	33	37	39	131	298	321	418
fexA01	G	A	L	I	V	P	A
fexA03	G	A	L	V	V	P	A
fexA05	G	A	S	I	M	P	A
fexA19	G	A	S	V	M	P	S
fexA17	**A**	**V**	L	V	V	**T**	A
fexA28	**A**	**V**	S	V	V	**T**	A

A. Minimum inhibitory concentrations (MICs, in mg/L) as assessed by microbroth dilution assays. B. Amino acid differences in the six *fexA* variants. Amino acids in red denote amino acid substitutions that are unique for the *fexA17* and *fexA28* variants.

Supplementary Figure 1.

Mutation and deletion in the *cfr* genes of isolates H5 and H7.

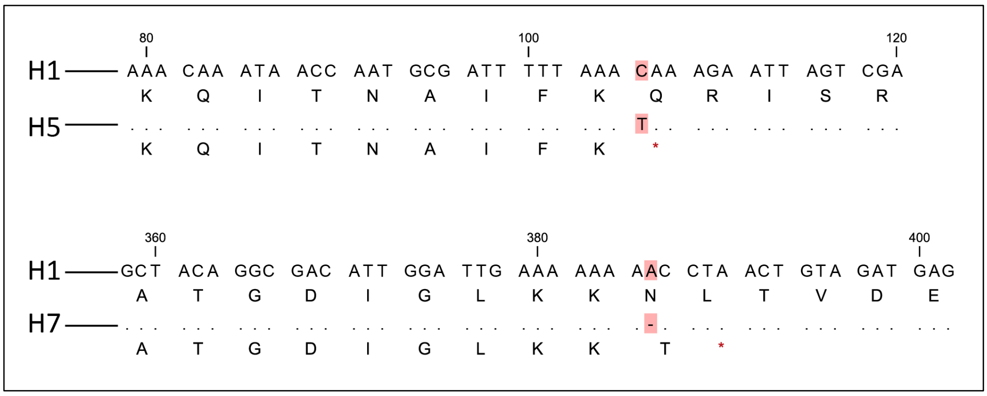

The mutation in isolate H5 results in the alteration of a glutamine (Q) codon into a stop codon. The deletion in isolate H7 results in a frameshift and a premature stop of the translation.

Supplementary Figure 2.

Pairwise wgMLST allelic distances between *cfr*-positive isolates.

A.

	H2	H3	H5	H7	H4	H6	P1	H1
H2	0							
H3	41	0						
H5	74	80	0					
H7	110	116	81	0				
H4	114	119	120	133	0			
H6	126	134	124	144	135	0		
P1	182	184	184	202	207	219	0	
H1	181	183	181	200	205	215	132	0

B.

	Number of alleles
Average distance	115
Median distance	125
Minimum distance	41
Maximum distance	219

A. Allelic distance matrix of *cfr*-positive isolates. B. Average, median, minimum, and maximum number of allelic distances among the eight cfr-positive isolates.

Supplementary Figure 3.

Insertion of the Tn*554* family transposons Tn*558* (*fexA*) and Tn*559* (*dfrK*) are inserted in the chromosomally located *radC* gene or in plasmids.

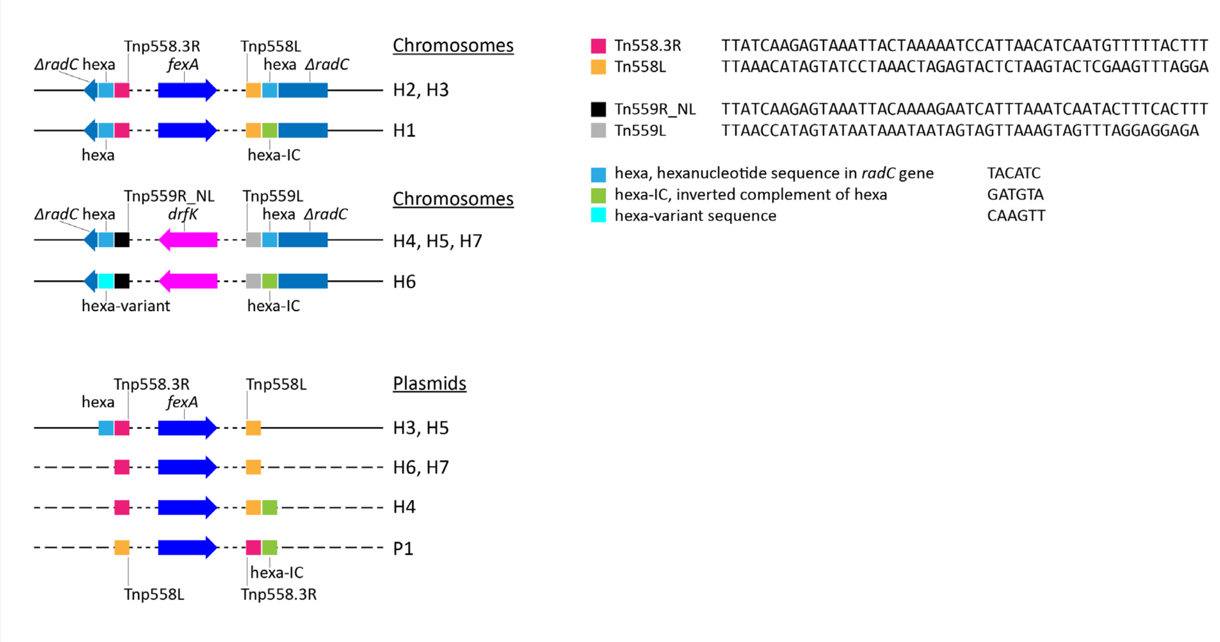

Supplementary Figure 4.

Pairwise sequence distance matrix between fexA sequence variants.

	fexA01	*fexA02*	*fexA03*	*fexA04*	*fexA05*	*fexA06*	*fexA07*	*fexA08*	*fexA09*	*fexA10*	*fexA11*	*fexA12*	*fexA13*	*fexA14*	*fexA15*	*fexA16*	*fexA17*	*fexA19*	*fexA20*	*fexA21*	*fexA22*	*fexA23*	*fexA24*	*fexA25*	*fexA27*	*fexA28*	*fexA29*	*fexA30*
fexA02	1/1																											
fexA03	1/1	2/2																										
fexA04	1/1	2/2	2/2																									
fexA05	2/2	3/3	3/3	1/1																								
fexA06	2/2	3/3	1/1	3/3	4/4																							
fexA07	2/2	3/3	1/1	3/3	4/4	2/2																						
fexA08	2/2	3/3	1/1	3/3	4/4	2/2	2/2																					
fexA09	2/2	3/3	3/3	3/2	4/3	4/4	4/4	4/4																				
fexA10	2/2	3/3	1/1	3/3	4/4	2/2	2/2	2/2	4/4																			
fexA11	3/3	4/4	2/2	4/4	5/5	3/3	3/3	1/1	5/5	3/3																		
fexA12	3/3	4/4	2/2	4/4	5/5	3/2	3/3	3/3	5/5	3/3	4/4																	
fexA13	3/3	4/4	2/2	2/2	3/3	3/3	3/3	1/1	5/4	3/3	2/2	4/4																
fexA14	3/3	4/4	2/2	4/4	5/5	1/1	3/3	3/3	5/5	3/3	4/4	4/3	4/4															
fexA15	3/3	4/4	2/2	2/2	3/3	3/3	3/3	3/3	5/4	3/3	4/4	4/4	2/2	4/4														
fexA16	4/4	5/5	3/3	5/5	6/6	4/4	4/4	2/2	6/5	4/4	1/1	5/5	3/3	5/5	5/5													
fexA17	4/4	5/5	3/3	5/5	6/6	4/3	4/4	4/4	6/6	4/4	5/5	1/1	5/5	5/4	5/5	6/6												
fexA19	4/4	5/5	3/3	3/3	2/2	4/4	4/4	4/4	6/5	4/4	5/5	5/5	3/3	5/5	3/3	6/6	6/6											
fexA20	4/3	5/4	3/2	3/2	4/3	4/3	4/3	2/1	6/4	4/3	3/2	5/4	1/0	5/4	3/2	4/3	6/5	4/3										
fexA21	4/4	5/5	3/3	5/5	6/6	4/4	4/4	2/2	5/5	4/4	1/1	5/5	3/3	5/5	5/5	2/0	6/6	6/6	4/3									
fexA22	4/4	5/5	3/3	3/3	4/4	4/4	4/4	2/2	6/5	4/4	3/3	5/5	1/1	5/5	3/3	4/4	6/6	4/4	2/1	4/4								
fexA23	5/5	6/6	4/4	4/4	3/3	5/5	5/5	3/3	7/6	5/5	2/2	6/6	2/2	6/6	4/4	3/3	7/7	3/3	3/2	3/3	3/3							
fexA24	5/4	6/5	4/3	6/5	7/6	5/3	5/4	5/4	7/6	5/4	6/5	2/1	6/5	6/4	6/5	7/6	1/0	7/6	7/5	7/6	7/6	8/7						
fexA25	5/5	6/6	4/4	6/6	7/7	5/4	5/5	5/5	7/7	5/5	6/6	2/2	6/6	6/5	6/6	7/7	1/1	7/7	7/6	7/7	7/7	8/8	2/1					
fexA27	5/5	6/6	4/4	6/6	7/7	5/4	5/5	5/5	7/7	5/5	6/6	2/2	6/6	6/5	6/6	7/7	1/1	7/7	7/6	7/7	7/7	8/8	2/1	2/2				
fexA28	5/5	6/6	4/4	4/4	5/5	5/4	5/5	5/5	7/6	5/5	6/6	2/2	4/4	6/5	4/4	7/7	1/1	5/5	5/4	7/7	5/5	6/6	2/1	2/2	2/2			
fexA29	5/5	6/6	4/4	6/6	7/7	5/4	5/5	5/5	7/7	5/5	6/6	2/2	6/6	6/5	6/6	7/7	1/1	7/7	7/6	7/7	7/7	8/8	2/1	2/2	2/2	2/2		
fexA30	5/4	6/5	4/3	6/5	7/6	5/3	5/4	5/4	7/6	5/4	6/5	2/2	6/5	6/4	6/5	7/6	3/3	7/6	7/5	7/6	7/6	8/7	4/3	4/4	4/4	4/4	4/4	
fexA34	19/8	20/9	18/7	20/8	21/9	19/8	19/8	19/8	19/8	19/8	20/9	20/9	20/8	20/9	20/8	21/10	21/10	21/9	21/8	21/10	21/9	22/10	22/10	22/11	22/11	22/10	22/11	22/10

The matrix displays the number of nucleotide differences and the number of resulting amino acid differences between variants. Nucleotide differences and amino acid differences are separated by a slash. The *fexA01* and *fexA02* sequences are extracted from the ResFinder database and originate from NCBI accession number AJ549214 and AM408573, respectively. The salmon-colored cells indicate *fexA* nucleotide sequence variants encoding for the same protein.
